# The assessment of returning to work following treatment and the associated personal, disease, and treatment factors among breast cancer survivors in central China

**DOI:** 10.1007/s00520-021-06354-y

**Published:** 2021-06-16

**Authors:** Min Li, Jinnan Gao, Ming Li, Linying Wang

**Affiliations:** 1 Department of Breast Surgery, Shanxi Bethune Hospital, Shanxi Academy of Medical Sciences, Tongji Shanxi Hospital, Third Hospital of Shanxi Medical University, Taiyuan, 030032 China; 2 Tongji Hospital, Tongji Medical College, Huazhong University of Science and Technology, Wuhan, 430030 China; 3 Cancer Research Institute, University of South Australia, Adelaide, 5000 Australia

**Keywords:** Breast cancer, Returning to work, Associated factors, Following treatment, China

## Abstract

**Purpose:**

To assess the status of returning to work (RTW) following breast cancer treatment and to explore its associated factors among female patients.

**Methods:**

Four-hundred-forty-two eligible patients admitted in a tertiary hospital since 2012 were followed up in 2018. Information about working status after treatment, date of RTW or reason for not RTW was obtained during a 30-min interview. Patients’ sociodemographic, disease, and treatment characteristics were retrieved from the hospital record. Overall prevalence rate and probability of RTW during the follow-up were estimated using Kaplan–Meier method. Factors associated with RTW were identified using regression analyses.

**Results:**

Three-hundred-ninety-six patients (89.6%) completed the follow-up. The median follow-up was 31 months. Among them, 141 patents (35.6%) RTW of whom 68.1% (n = 96) were back within 12 months after cancer treatment. The reported reasons for not RTW included: prolonged fatigue, low self-esteem, lack of support from family and working unit, or voluntarily quitting. Patients aged under 50 years, being single, having higher level of education, not having extensive axillary node procedure, or without any comorbidities were more likely to RTW.

**Conclusion:**

The rate of RTW after cancer treatment in this cohort was lower than those reported in others. Both personal and treatment factors were associated with RTW.

## Introduction

Breast cancer has become the most common malignancy in females in China. It was estimated that the age-standardized incident rate was 36.1/10^5^ in 2018, accounting for nearly 20% of all cancer incidence [1]. The increased incidence of breast cancer tended to occur at younger age and patients younger than 50 years took up about 57% of all cases [2]. Owing to the continuous innovation and development of diagnosis and treatment technology, the 5-year relative survival increased from 72% in 2003 to 82% in 2015 [3]. Challenges for patients and their family, and health care providers arise regarding how to best improve younger patients’ long-term mental health and quality of life after treatment. Returning to work (RTW) and engaging in the community have been shown as being beneficial to patients’ physical and mental health and having an important impact on patients’ overall life satisfaction [4]. In addition, RTW would not only reduce the economic burden of the family and elevate self-perception, but also promote the patient’s return to the society and achieve vocational rehabilitation [5].

Studies have shown that the prevalence of the RTW varies from 43 to 93% within one year of diagnosis in European and North American countries [6]. The lowest was in The Netherlands of a 43% after 390 days of medical leave [7], while US survivors had the highest rate of a 93% within 12 months following diagnosis and 67% were continuously working five to seven years after diagnosis [8]. Factors associated with RTW included socio-demographic factors (i.e., age, marital status, number of children, family income, ethnicity), disease-related factors (such as stage of disease, comorbidities), treatment-related factors, psychological factors (e.g., coping skills, life satisfaction), work-related factors (including work demands, flexibility of work schedule, work environment), policies and economic factors [9].

Yet, study about RTW and its associated factors have not been assessed in China. Further investigation of its associated factor could provide cultural-specific evidence to help breast cancer survivors in China. We aimed to fill the knowledge gap in this study to assess the status of RTW after treatment and its sociodemographic, disease, and treatment factors associated among a cohort of women with breast cancer in central China.

## Methods

### Study population

A cohort of 442 eligible patients with breast cancer admitted in the Breast Surgery Department at a tertiary hospital in Shanxi Province during 2012–2018 were included in this study. The eligibility criteria included: diagnosed with breast cancer without distance metastasis; having a job at the diagnosis; aged under the national age cut off for retirement (maximum 55 years); being able to communicate efficiently; having consented to participate in this study; having completed records in the hospital database (demo-socioeconomic, disease, and health information). The patients were initially approached via phone or a social app (WeChat), and were informed of the study purpose, procedure, privacy protection, and data storage. A 30-min phone interview or scheduled hospital follow-up was booked or confirmed if they consented in written to proceed. Patients could quit interview at any time even if they consented to participate the study. The study proposal was reviewed and approved by the hospital ethics committee (approval No. YXLL-2020–027).

### Study outcome

RTW was defined in this study as the status of going back working as usual before cancer diagnosis. The following information were collected during the interview: status of RTW, the date of RTW, and whether the job was the same as the one at diagnosis, or new job if changed; and the reason(s) if not RTW. Interviews were conducted by trained research staff from the Breast Surgery Department in 2018 based on a standardized interview guide.

### Study factors

The following information was obtained from the hospital registry database: demo-socioeconomic (age at diagnosis, education attainment, occupation, marital status, health insurance coverage); cancer profile (tumor size, cancer stage, laterality), cancer treatment (surgery type, chemotherapy, radiotherapy, targeted therapy, endocrinal therapy, axillary node procedure), patients’ health conditions at diagnosis (for example, diabetes, hypertension, cardiovascular conditions, thyroid conditions, gynecological conditions, and others), from which the Charlson Comorbidity Index was composed [10] and broadly categorized as 0 and ≥ 1 due to small numbers for index score ≥ 2.

### Statistical analysis

Follow-up time (in month) was from the date completing treatment to the date of RTW or to the interview date for patients not RTW. Patients’ and disease characteristics were compared by follow-up status and by RTW status with Chi-square test. The probability of RTW following treatment was estimated with Kaplan–Meier method. The study factors associated with RTW were investigated and identified using stepwise Cox regression analysis after proportional hazard assumption was justified. Cox analysis evaluated all follow-up time. Variables with P < 0.10 from univariate analysis were initially included in a full model and dropped each at a time justified by likelihood ratio test. In addition, multivariate generalized linear regression analysis was conducted with family being “binomial” and link function being “log” to avoid biased association estimates since the prevalence rate of RTW was not rare (35.6%) for logistic regression analysis [11]. All the analyses were performed using STATA 14.2 (Stata Corporation, College Station, USA). Statistical significance was considered when P < 0.05 (two-sided).

## Results

**Fig. 1:**
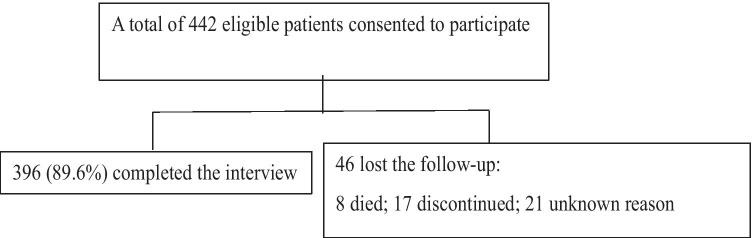
Description of the cohort of breast cancer survivors during 2012–2018

Among the 442 patients, 396 (89.6%) completed the interview. Of the 46 who discontinued the follow-up, eight died and 17 patients changed mind, and 21 patients missed interview without any reason(s) (Fig. 1). They were comparable in patients’ and disease characteristics to those who completed the follow-up (Table 1).

**Table 1:** Comparison of demo-socioeconomic and disease profile by follow-up status among 442 breast cancer patients in 2012–2018

	Completed follow-up (n = 396)	Loss of follow-up (n = 46)	*X*^*2*^	*p*
Age			2.311	0.128
< 50	316 (79.8%)	41 (89.1%)		
> 50	80 (20.2%)	5 (10.9%)		
Education attainment			5.069	0.079
< Junior High	169 (42.7%)	16 (34.8%)		
Senior high school or equivalent	94 (23.7%)	7 (15.2%)		
> University	133 (33.6%)	23 (50.0%)		
Occupation			1.928	0.588
Factory worker	71 (17.9%)	12 (26.1%)		
Office clerk	129 (32.6%)	14 (30.4%)		
Management and academia	100 (25.3%)	11 (23.9%)		
Other	96 (24.2%)	9 (19.6%)		
Marital status (married)	374 (94.4%)	43 (93.5%)	0.072	0.788
% Medical insurance coverage			0.714	0.700
0	159 (39.9%)	16 (34.8)		
50–60%	74 (18.7%)	8 (17.4%)		
70–90%	164 (41.4%)	22 (47.8%)		
Tumour laterality			0.737	0.391
Right	190 (48.0%)	19 (41.3%)		
Left	206 (52.0%)	27 (58.7%)		
TNM staging			0.757	0.860
0	26 (6.6%)	2 (4.3%)		
I	132 (33.3%)	14 (30.4%)		
II	182 (46.0%)	22 (47.8%)		
III	56 (14.1%)	8 (17.4%)		
Targeted therapy (yes)	57 (14.4%)	8 (17.4%)	0.295	0.587
Radiotherapy (yes)	271 (68.4%)	32 (69.6%)	0.024	0.876
Chemotherapy (yes)	326 (82.3%)	37 (80.4%)	0.100	0.752
Endocrine therapy (yes)	239 (60.4%)	26 (56.5%)	0.252	0.616
Surgery type			0.271	0.603
Mastectomy	187 (47.2%)	23 (50.0%)		
Conserving	209 (52.8%)	23 (50.0%)		
Axillary node procedure			2.172	0.141
Sentinel node biopsy	209 (52.8%)	19 (41.3%)		
Node dissection	187 (47.2%)	27 (58.7%)		
Charlson Comorbidity Index ≥ 1	147 (37.1%)	15 (32.6%)	0.688	0.407

Numbers in the table were n (%); p from Chi-square test

Of those who completed the follow-up, the median age was 45 years. 57% attained at least senior high schooling, around a quarter were managers or teachers (lecturers), 94% were married and 60% had medical insurance. 86% had tumors of stage 0–II, 53% underwent breast conserving surgery. 37% had one or more commodities indicated by Charlson Comorbidity Index ≥ 1 (Table 1).

Compared to those not RTW (n = 255), patients who RTW (n = 141) were: younger, having higher level of education, or working in management or academia sector at diagnosis, not married, having better health insurance coverage, or having no comorbidity (Charlson Comorbidity Index of 0) (Table 2). After adjusted for factors of P < 0.10 in the multivariate model, the factors significantly associated with RTW were age, education, and Charlson Comorbidity Index. Specifically, women aged under 50 years were twice more likely to RTW (aOR 1.97, 95% CI 1.12–3.45) compared to those over 50; the adjusted odds (95% CI) of RTW was 1.96 (1.13, 3.37) for those completed senior high and 2.61 (1.53, 4.45) for university graduates compared to those who attained junior high education; women with no comorbidity were five times more likely to RTW (aOR 5.32, 95% CI 2.74–10.35) compared to those with Charlson Comorbidity Index ≥ 1 (Table 2).

**Table 2:** Patients’ characteristics, cancer profile and treatment by RTW status among patients with breast cancer in 2012–2018 (n = 396)

	Not RTW (n = 255)	RTW (n = 141)	p	aOR (95% CI)
Age (years)			< 0.001	
> 50	66 (25.9%)	14 (9.9%)		1.0
< 50	189 (74.1%)	127 (90.1%)		1.97 (1.12, 3.45)
Education attainment			< 0.001	
< Junior school	144 (56.5%)	25 (17.7%)		1.0
Senior school or equivalent	63 (24.7%)	31 (22.0%)		1.96 (1.13, 3.37)
> University	48 (18.8%)	85 (60.3%)		2.61 (1.53, 4.45)
Occupation			< 0.001	
Office clerk	91 (35.7%)	38 (27.0%)		1.0
Factory worker	48 (18.8%)	23 (16.3%)		1.15 (0.67, 1.97)
Management and academia	41 (16.1%)	59 (41.8%)		1.32 (0.86, 2.02)
Other	75 (29.4%)	21 (14.9%)		1.14 (0.66, 1.97)
Marital status			0.018	
Married	246 (96.5%)	128 (90.8%)		1.0
Not married	9 (3.5%)	13 (9.2%)		1.15 (0.64, 2.06)
% of medical insurance coverage			< 0.001	
0	105 (41.2%)	53 (37.6%)		1.0
50–60%	62 (24.3%)	12 (8.5%)		1.18 (0.48, 2.90)
70–90%	88 (34.5%)	76 (53.9%)		1.51 (0.65, 3.52)
Tumour laterality			0.777	
Right	121 (47.5%)	69 (48.9%)		
Left	134 (52.5%)	72 (51.1%)		
TNM staging			0.132	
0	19 (7.5%)	7 (5.0%)		
I	75 (29.4%)	57 (40.4%)		
II	125 (49.0%)	57 (40.4%)		
III	36 (14.1%)	20 (14.2%)		
Targeted therapy			0.088	
No	224 (87.8%)	115 (81.6%)		1.0
Yes	31 (12.2%)	26 (18.4%)		1.04 (0.67, 1.61)
Radiation therapy			0.214	
No	86 (33.7%)	39 (27.7%)		
Yes	169 (66.3%)	102 (72.3%)		
Chemotherapy			0.092	
No	42 (16.5%)	33 (23.4%)		1.0
Yes	213 (85.5%)	108 (76.6%)		0.83 (0.56, 1.22)
Endocrine therapy			0.139	
No	108 (42.2%)	49 (34.8%)		
Yes	147 (57.6%)	92 (65.2%)		
Surgery type			0.241	
Mastectomy	126 (60.0%)	61 (43.3%)		
Conserving	129 (40.0%)	80 (56.7%)		
Axillary node procedure			0.071	
Sentinel node biopsy	126 (49.4%)	83 (58.9%)		1.0
Node dissection	129 (50.6%)	58 (41.1%)		1.11 (0.79, 1.57)
Charlson Comorbidity Index			< 0.001	
> 1	137 (53.7%)	10 (7.1%)		1.0
0	118 (46.3%)	131 (92.9%)		5.32 (2.74, 10.35)

*RTW*, returning to work; Numbers in the table were n (%); p from Chi-square test; *aOR*, adjusted odds ratio from generalized linear regression analysis of the variables with p < 0.10 with family being “binomial” and link function being “log” to avoid biased association since the RTW rate was not rare [11]

**Fig. 2:**
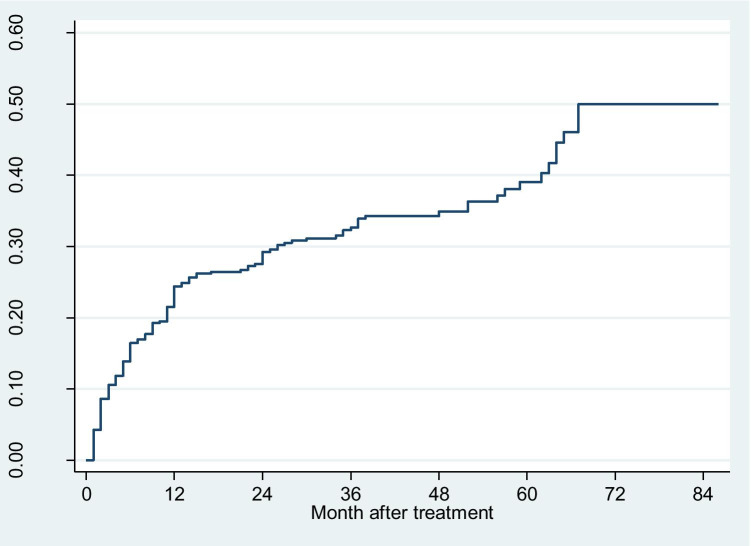
Probability of RTW in breast cancer patients diagnosed during 2012–2018

Of those who completed the interview in a median follow-up of 31 months (minimum 1 month, maximum 86 months, inter-quartile range 12–50 months), 141 patients RTW in an average of 15 months after treatment (median 9 months, inter-quartile range 3–22 months). 17 RTW within one month, 96 RTW within 12 months, and 88% (n = 124) were back at the 36 months following the completion of treatment. Figure 2 showed the probability of RTW during the follow-up period. For example, at the end of the first month following treatment, 17 patients RTW among 396 patients, given a probability of 4.3% (17/396). The probability of RTW was 39.0% at the 60th-month and 50% at the 67th-month till the end of follow-up. The overall RTW rate was 11.0 per 1000 person-month (9% CI 9.3–12.9 per 1000 person-month). Of those RTW, 45 were back to the previous position while 96 women changed position or reduced working hours and/or workload. Of the 255 patients failing to RTW at the time of interview, 51 (20%) reported low-esteem, 62 women (24.3%) felt prolonged fatigue, 44 (17.2%) reported lacking of support from supervisors or colleagues or even being discriminated, 39 women chose to quit, 38 reported being difficult to concentrate on work, while 21 (8.3%) reported being prevented from family.

The probability of RTW at the 12th- and 36th-month following cancer treatments were presented in Table 3. The overall probabilities of RTW at the two time points were 24.4% (95% CI 20.4%, 28.9%) and 32.7% (95% CI 28.2%, 37.7%), respectively. Women under 50 years or being single were 2.7 times (unadjusted HR 2.66, 95% CI 1.53–4.63) or 2.5 times (HR 2.45, 95% CI 1.38, 4.34) more likely to RTW compared to the corresponding counterparts. Those with senior high or university degree were twice (HR 2.36, 95% CI 1.39–4.00) or seven times (HR7.25, 95% CI 4.61–11.39) more likely to RTW compared to those with junior high education; women working in the management and academic sector were twice more likely to RTW (HR 2.65, 95% CI 1.76–3.98) compared to officer clerk at diagnosis. Compared to patients with lower medical insurance coverage, those with higher coverage were four times more likely to RTW (HR 4.10, 95% CI 2.23–7.55). Patient with no comorbidities (Charlson Comorbidity Index of 0) or having targeted therapy or no radical axillary node surgery was associated with increased likelihood of RTW (Table 3).

**Table 3:** Probability (%) of RTW (95 CI) at 12th-, 36th-month and the associated factors (HR, 95% CI) among 396 patients diagnosed in 2012–2018

	At 12th month (95% CI)	At 36th month (95% CI)	P*	Unadjusted HR (95% CI)	Adjusted HR (95% CI)**
Age (years)			0.003		
> 50	11.3 (6.0, 20.5)	18.5 (11.4, 29.4)		1.0	1.0
< 50	27.7 (23.1, 33.0)	36.3 (31.1, 42.1)		2.66 (1.53, 4.63)	2.57 (1.47, 4.49)
Education attainment			< 0.001		
< Junior school	7.1 (4.1, 12.2)	11.7 (7.5, 18.0)		1.0	1.0
Senior high or equivalent	16.1 (10.0, 25.2)	25.9 (18.0, 36.5)		2.36 (1.39, 4.00)	2.07 (1.22, 3.50)
> University	52.4 (44.2, 61.2)	64.3 (55.9, 72.6)		7.25 (4.61, 11.39)	5.16 (3.25, 8.18)
Occupation			< 0.001		
Office clerk	17.1 (11.6, 24.9)	26.5 (19.5, 35.5)		1.0	
Factory worker	21.4 (13.5, 33.0)	28.3 (19.0, 40.8)		1.10 (0.65, 1.84)	
Management and academia	51.3 (41.9, 61.5)	59.0 (49.4, 68.8)		2.65 (1.76, 3.98)	
Other	8.3 (4.3, 16.0)	16.8 (10.4, 26.4)		0.69 (0.40, 1.17)	
Marital status			0.001		
Married	22.6 (18.7, 27.2)	31.1 (26.5, 36.3)		1.0	1.0
Not married	54.6 (35.7, 75.6)	59.1 (39.9, 79.0)		2.45 (1.38, 4.34)	1.85 (1.03, 3.31)
% of medical insurance coverage			< 0.001		
50–60%	4.1 (1.3, 12.0)	12.7 (6.5, 24.0)		1.0	
0	21.7 (16.0, 29.0)	27.2 (20.9, 35.0)		2.53 (1.35, 4.74)	
70–90%	36.2 (29.4, 44.1)	47.1 (39.5, 55.4)		4.10 (2.23, 7.55)	
Tumour laterality			0.817		
Right	24.4 (18.9, 31.2)	33.7 (27.2, 41.2)		1.0	
Left	24.4 (19.1, 30.9)	31.8 (25.7, 38.8)		0.96 (0.69, 1.34)	
TNM staging			0.113		
0	23.1 (11.1, 44.3)	27.9 (14.3, 50.0)		1.0	
I	32.8 (25.5, 41.6)	42.0 (34.0, 51.1)		1.79 (0.82, 3.92)	
II	19.9 (14.8, 26.5)	26.5 (20.5, 33.8)		1.13 (0.51, 2.48)	
III	19.7 (11.4, 32.8)	32.9 (21.7, 47.9)		1.30 (0.55, 3.07)	
Targeted therapy			0.027		
No	22.2 (18.2, 27.1)	30.5 (25.7, 35.9)		1.0	
Yes	37.2 (26.0, 51.1)	46.3 (33.8, 60.9)		1.60 (1.05, 2.46)	
Radiation therapy			0.457		
No	21.0 (14.8, 29.3)	30.9 (23.0, 40.6)		1.0	
Yes	25.9 (21.1, 31.6)	33.7 (28.3, 39.8)		1.15 (0.79, 1.66)	
Chemotherapy			0.044		
No	29.4 (20.5, 41.1)	45.3 (34.3, 57.9)		1.0	
Yes	23.2 (18.9, 28.2)	29.7 (24.9, 35.2)		0.67 (0.46, 1.00)	
Endocrine therapy			0.258		
No	22.0 (16.2, 29.4)	30.1 (23.2, 38.5)		1.0	
Yes	26.0 (20.9, 32.0)	34.4 (28.7, 41.0)		1.22 (0.86, 1.72)	
Surgery type			0.113		
Mastectomy	17.2 (12.5, 23.5)	29.4 (23.1, 36.9)		1.0	
Conserving	30.7 (24.9, 37.5)	35.6 (29.4, 42.6)		1.30 (0.93, 1.82)	
Axillary node procedure			0.029		
Node dissection	17.8 (13.0, 24.1)	26.8 (20.8, 34.2)		1.0	1.0
Sentinel node biopsy	30.2 (24.5, 37.0)	37.9 (31.6, 45.1)		1.45 (1.03, 2.02)	1.41 (1.01, 1.99)
Charlson Comorbidity Index			< 0.001		
> 1	1.4 (0.3, 5.3)	4.6 (2.1, 9.9)		1.0	1.0
0	38.1 (32.4, 44.5)	49.9 (43.5, 56.6)		10.68 (5.60, 20.35)	7.75 (4.04, 14.86)

^*^P from log-rank test

^**^adjusted HR (95% CI) from the final stepwise Cox regression analysis by initially including variables with P < 0.1 from univariate analysis; variables no longer significantly associated with RTW was eliminated each at one time justified with likelihood ratio test; The factors subsequently eliminated from the model were: hormone therapy, occupation, surgery type, cancer stage, cancer side, target therapy, insurance coverage, radiotherapy. The final model included age at diagnosis, education attainment, marital status, axillary surgery status, and comorbidities at diagnosis

In the stepwise analysis, factors were subsequently dropped included hormone therapy, occupation, breast surgery type, cancer stage, tumor laterality, targeted therapy, insurance coverage, and radiotherapy. Factors retained in the final model included: age at diagnosis (adjusted HR 2.57, 95% CI 1.47, 4.49 for aged under 50 years), education attainment (aHR 2.07, 95% CI 1.22–3.50 for senior high and 5.16, 95% CI 3.25–8.18 for university attainment), marital status (aHR 1.85 95% CI 1.03–3.31 for being single), axillary surgery status (aHR 1.41, 95% CI 1.01–1.99 for sentinel node biopsy), and comorbidities at diagnosis (aHR 7.75, 95% CI 4.04–14.86 for Charlson Comorbidity Index of 0) (Table 3).

Both Cox regression and the generalized linear regression identified significant factors such as age, education, and comorbidities, while the Cox regression picked out two additional factors including marital status and axillary node procedure. Further exploration indicated the likelihood of RTW was time-dependent, supporting the findings from the Cox regression analysis.

## Discussion

This was the first assessment of RTW status in a median of 31-month follow-up of 442 female breast cancer patients admitted in a tertiary hospital in central China during 2012–2018. Predicting factors of RTW included not only patient’s and family factors, but also diseases and its treatment as well. We have explored reasons for not RTW. The results is of great significance in that breast cancer patients in China were 10 years younger with the median age at diagnosis of 50–54 compared to 55–59 in the USA and European Union [12] and 57% of them were under 50 years [2]. Our hospital data showed during the diagnosis period of 2012–2018, 87.5% of the 551 women aged under 55 years were employed at diagnosis, which may be lower than in the big cities in China and is not representative to the national level. We estimate a larger proportion of breast cancer patients under 55 years would have to decide whether to RTW during or after treatment. The results of this study can facilitate to understand patients’ reflections on RTW, and to develop targeted strategies to promote patients’ recovery, quality of life, and return to the society as normal in the short term and in the long run.

The overall prevalence of RTW was 36% and 88% of those who were able to RTW within 36 months following treatment. The probability of RTW at the 12th- and 36th months were 24.4% and 32.7%, respectively. This level of RTW was lower than the prevalence of 43 to 93% reported in other countries, e.g. The Netherlands, Sweden, and USA, within one year of diagnosis [6–8], although direct comparison was implausible due to variations in recorded time for RTW started from the completion of cancer treatment. The RTW rate was lower than the rate reported in Brazilian breast cancer survivors of a 54.0% after treatment [13]. The gap was mainly due to more advanced disease stag [14]. It should be noted that the retiring age for woman in China is 50 for blue collar and 55 for white collar workers to be eligible to access to social security payment under the current legislation from Chinese Academy of Labor and Social Security of the Ministry of Human Resources and Social Security. Based on the National Classification of Occupation in China, blue collar workers are referred to manual workers or heavy laborers or machine operators in industrial sectors such as factory, farming, forestry, fishery, transportation, and in business and service sectors; while white collar workers are public servants, administrators and officer clerks, professional staff [15]. The Labor Law of China regulates the sick leave entitlements for employees (at least 80% of the salary) and flexible options to continue to work or to retire early based on medical advice, patient’s preference, and employer’s needs.

We found in this cohort that age was a strong predictor of RTW after treatment, consistent with the systematic review including studies in European and American [6]. The results reflected the need of social life and work among younger patients in one hand, requiring health care providers to pay attention to their psychological status, and to help or guide them to reorient their life and work after the traumatized cancer treatment.

Patients with higher education were more likely to RTW, as reported by others [16]. This subgroup has better working conditions and environment, and they are mostly the technical backbones, or at management level in the working unit, and have certain control over their work; in addition, they are more competitive to get new jobs [17].

Thirty-nine patients (15.3%) in this cohort chose to change their career and spent more time with family after being diagnosed with breast cancer. Some valued working essential for life and a sign of recovery. Patients also reported different levels of shock, trauma, stress, and low self-esteem at diagnosis, during and/or after treatment, which leads to psychological and mental disturbance and reluctance to RTW [18].

We found that 21 cases (8.3%) were unable to RTW due to family intervention which is consistent with the study of Cocchiara et al. [19]. Family support can help patients effectively integrate various resources, enable them to adapt to any difficulties, and promote the recovery of mental health [20]. Medical and health care team cannot ignore family support in patient management during treatment, recovery and RTW. On the other hand, our data showed that married women were less likely to RTW, which might indicate less family financial burden when family were supportive and did not put any financial stress on them [21].

Some patients in this cohort sought for professional occupational rehabilitation guidance. 62 patients (24.3%) reported feeling tired, and 38 (14.9%) unable to concentrate. The results were consistent with the report indicating that these symptoms after treatment affected their ability to conduct a work [22]. We also found that patients with comorbidities were less likely to RTW, similar to the studies reporting that patients with fatigue, pain, or cognitive decline, choose to avoid social interaction, and therefore seriously affecting their ability to work and/or quality of life [23, 24]. It is necessary to formulate individual rehabilitation plans during or after treatment to restore their physical functions or mental wellbeing for work and social activities [25].

Of this cohort, 51 cases (20%) refused to work due to self-image concerns. We found that having radical axillary node surgery decreased the probability to RTW. The breast shape change after surgical treatment put extra stress on body image as studies have showed that the negative emotion such as tension, anxiety, and fear prevent women to participate in daily social activities [26, 27].

Among those failing to RTW, 44 (17.2%) patients reported lacking support from supervisors or colleagues or even being discriminated. Studies have shown that the nature of work, work intensity and environment, and prejudice against breast cancer patients have different degrees of impact on their RTW [28–30], while the support of the work unit plays a key role in promoting patients RTW [31, 32]. It is necessary for employers to provide flexible work arrangement and a friendly working environment so that patients can get a sense of belonging and sense of achievement from work.

This cohort study assessed the status of RTW after treatments and its associated factors in a cohort of 442 women with breast cancer during 2012–2018. The follow-up time lasted relatively long for a median 31 months. Loss of follow-up was rare and the studied characteristics of the subgroup did not differ from the completing cohort. Comprehensive multidimensional factors of sociodemographic and health conditions, cancer profile, and treatments were included in the investigation. The main associated factors were investigated using RTW as a single event, or a time-dependent event, and generally consistent with additional robust statistical justification. In addition, reasons for not RTW were discussed and explored with the patients to assist understanding of RTW status and the patients’ needs after treatment.

Limitations of this study should be noted. Firstly, this cohort is from one tertiary hospital in central China and the results may not be applied to other patients. Further study from other regions in China could be conducted to confirm our results; secondly, reasons for not RTW were self-reported and not tested statistically due to small sample size. Finally, common health conditions in this cohort such as diabetes, cardiovascular, thyroid, and gynecological conditions were not separately investigated rather collectively as comorbidities due to smaller case numbers.

## Conclusion

The prevalence of RTW was lower among female breast cancer survivors in central China than in other countries. Age at diagnosis, education attainment, node surgery, and health conditions (comorbidities) significantly predicted RTW. Reasons for not RTW were multidimensional, including patient’s factors such as disease and symptoms, family support, and health care services, and environmental support from wider community.

## Data Availability

The datasets analyzed during this study are available from the corresponding author on reasonable request.

## Code availability

Codes are available from the corresponding author on reasonable request.
